# Histological and Biomechanical Comparison of Different Dissection Planes in Brow Lift: An Experimental Rabbit Model and Its Clinical Implications

**DOI:** 10.1007/s00266-026-05873-5

**Published:** 2026-04-29

**Authors:** Serhat Şibar, Muzaffer Duran, Sami Can Yeşilırmak, Elifnaz Perdeci, Fatma Kübra Erbay Elibol, Süheyla Esra Özkoçer, Bilge Kaan İsmail

**Affiliations:** 1 Department of Plastic, Reconstructive & Aesthetic Surgery, Gazi University Faculty of Medicine, Ankara, Turkey; 2 Department of Biomedical Engineering, Faculty of Engineering, TOBB Economics and Technology University, Ankara, Turkey; 3 Department of Histology and Embryology, Faculty of Medicine, Gazi University, Ankara, Turkey; 4 Department of Plastic, Reconstructive & Aesthetic Surgery, Erzurum State Hospital, Erzurum, Turkey

**Keywords:** Biomechanical analysis, Brow-lift, Dissection plane, Histological evaluation, Subcutaneous approach

## Abstract

**Background:**

Different dissection planes used in brow lift surgery—namely, subcutaneous, subgaleal, and subperiosteal—present distinct biomechanical and histological characteristics. This study aimed to compare these three dissection approaches in terms of tissue healing and mechanical resistance.

**Methods:**

In this experimental model using New Zealand white rabbits, three groups of eight animals each underwent subcutaneous, subgaleal, or subperiosteal dissection. After a 12-week healing period, tissue samples were harvested for either histological or biomechanical analysis. Epidermal, dermal, superficial musculoaponeurotic system (SMAS), and periosteal thicknesses were quantitatively measured using ImageJ software. Biomechanical parameters assessed included maximum load, stiffness, and yield load.

**Results:**

Histological evaluation revealed preserved tissue integrity in all groups, with no evidence of fibrosis or disrupted collagen organization. A statistically significant difference in the epidermis/dermis ratio was observed between the control group and both the subgaleal and subperiosteal groups (*p *< 0.05). In biomechanical testing, the subperiosteal group demonstrated significantly superior values for maximum load, stiffness, and yield load compared to the other groups (*p *< 0.05).

**Conclusion:**

These findings suggest that while the subperiosteal plane offers greater long-term mechanical stability, the subcutaneous approach may support a more physiological healing pattern. This is supported by its closer resemblance to the control group in epidermis-to-dermis ratios and the absence of fibrotic remodeling. The preservation of native tissue architecture in this group highlights its potential as a biologically favorable plane in selected patients. Dissection plane selection should be individualized based on the patient’s specific tissue characteristics and surgical goals.

**No Level Assigned:**

This journal requires that authors assign a level of evidence to each submission to which Evidence-Based Medicine rankings are applicable. This excludes review articles, book reviews, and manuscripts that concern basic science, animal studies, cadaver studies, and experimental studies. For a full description of these Evidence-Based Medicine ratings, please refer to the Table of Contents or the online Instructions to Authors www.springer.com/00266.

## Introduction

Eyebrow position plays a key role in achieving a youthful and dynamic upper face. Age-related brow ptosis has led to various surgical techniques for forehead and periorbital rejuvenation [1–6]. Brow lift surgery has evolved since the early 20th century and is now performed in subcutaneous, subgaleal, subperiosteal, or combined planes [5, 7–10].

Each plane has advantages and limitations. The subperiosteal plane, often preferred in endoscopic approaches, provides a bloodless field, periosteal resistance to stress relaxation, and improved optical cavity [11, 12]. However, healing may take longer, and early postoperative stability can be limited. The subgaleal plane, traditionally associated with the coronal lift technique, is easy to dissect with relatively rapid recovery, but without periosteal release, brow elevation may be limited [8, 13–15].

Recently, the subcutaneous plane has regained popularity with techniques such as the gliding brow lift, offering superficial dissection, results aligned with aging anatomy, no need for osseous fixation, and low complication rates [5, 16, 17]. However, its histological and biomechanical healing has not been well investigated experimentally. In the literature, many animal studies focus on the subperiosteal plane, evaluating periosteal readhesion, fibrosis, and flap strength. Still, most have short follow-up, small sample sizes, and limited statistical analyses [13, 18–22]. The subcutaneous plane has often been excluded or not evaluated as a separate group.

Therefore, a controlled model comparing the subcutaneous, subgaleal, and subperiosteal planes under the same protocol is needed. This study compares their histological healing and biomechanical performance in a rabbit model and discusses potential clinical implications.

## Materials and Methods

This study was approved by the Animal Experiments Local Ethics Committee (G.U.ET-23.108). Twenty-seven female New Zealand white rabbits (12 weeks old, 2500–3000 g) were used. Subjects were randomly assigned to three experimental groups of eight animals each. The remaining three served as the control group and underwent no surgical procedure.

### Surgical Procedure

**Fig. 1:**
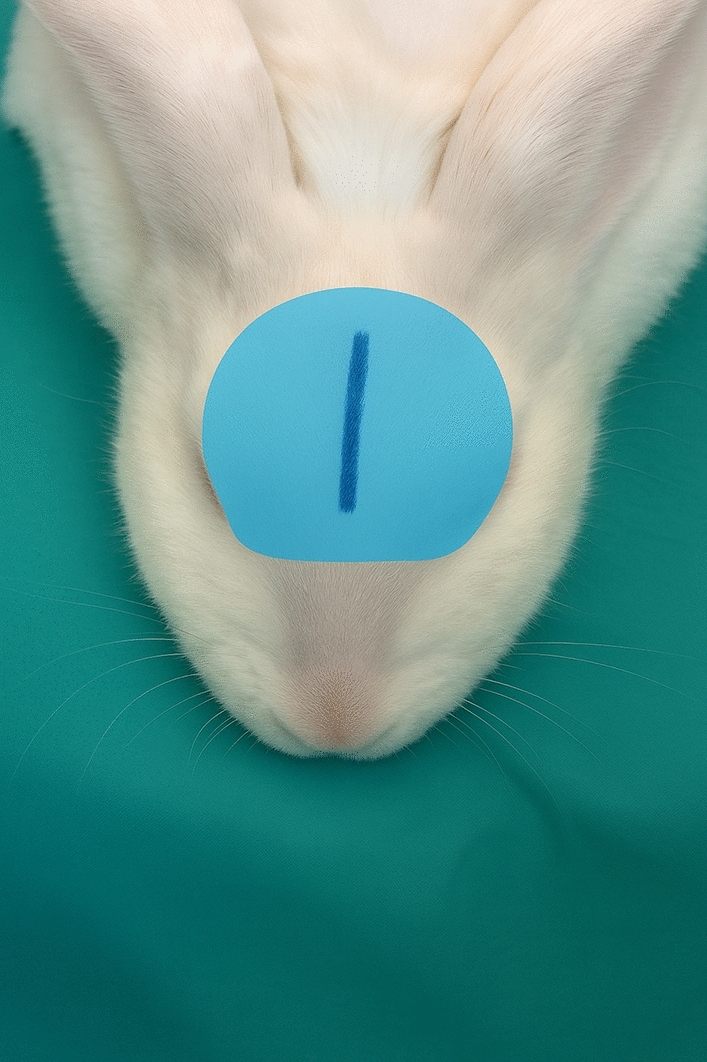
Digitally illustrated schematic representation of the planned midline incision and surgical dissection area in the frontal region

**Fig. 2:**
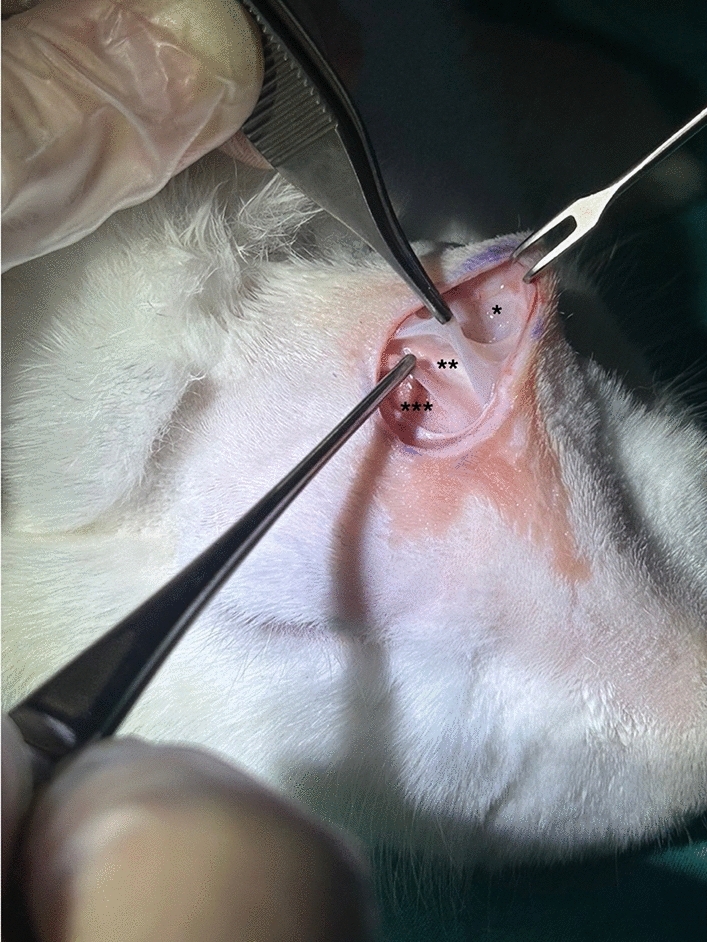
Intraoperative view of the planned dissection planes in the subjects. *Subcutaneous dissection plane (Group 1), **subgaleal dissection plane (Group 2), and ***subperiosteal dissection plane (Group 3) are indicated

All operations were performed under general anesthesia (intramuscular ketamine 45 mg/kg and xylazine 5 mg/kg). The frontal region was shaved and disinfected with povidone-iodine. A 3 cm vertical midline skin incision was made, and the area between the ears, orbital rims, and nasal radix was dissected according to the assigned plane (Figs. 1 and 2).

**Fig. 3:**
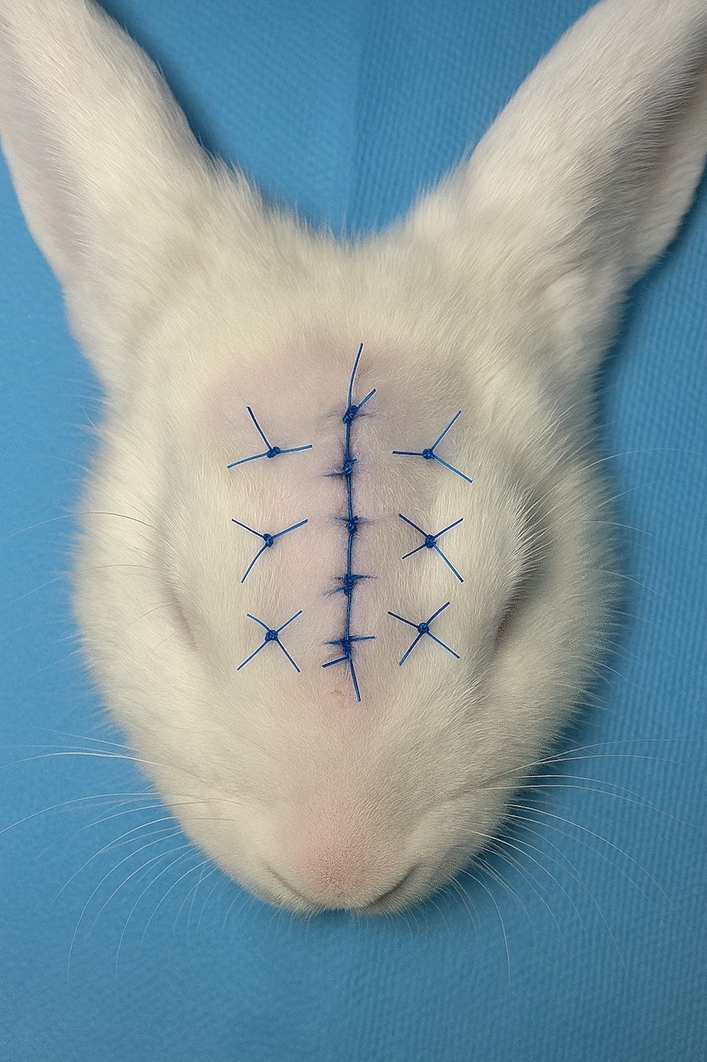
Digitally illustrated postoperative view showing subcutaneous dissection and surgical net sutures applied in Group 1

*Group 1 (Subcutaneous dissection)* For hydrodissection, 10 cc saline was infiltrated into the subcutaneous tissue before incision. Dissection was then performed in the subcutaneous plane with blunt-tipped tenotomy scissors. A 1 cm segment of skin was excised bilaterally. Closure was performed with 4-0 polyglactin for the subcutaneous layer and 5-0 polypropylene for the skin. On each side of the incision, three 5-0 polypropylene “surgical net” sutures were applied (Fig. 3).

*Group 2 (Subgaleal dissection)* Dissection was carried out in the subgaleal plane. After bilateral 1 cm skin excision, galeal tissue was closed with 4-0 polyglactin and the skin with 5-0 polypropylene sutures.

**Fig. 4:**
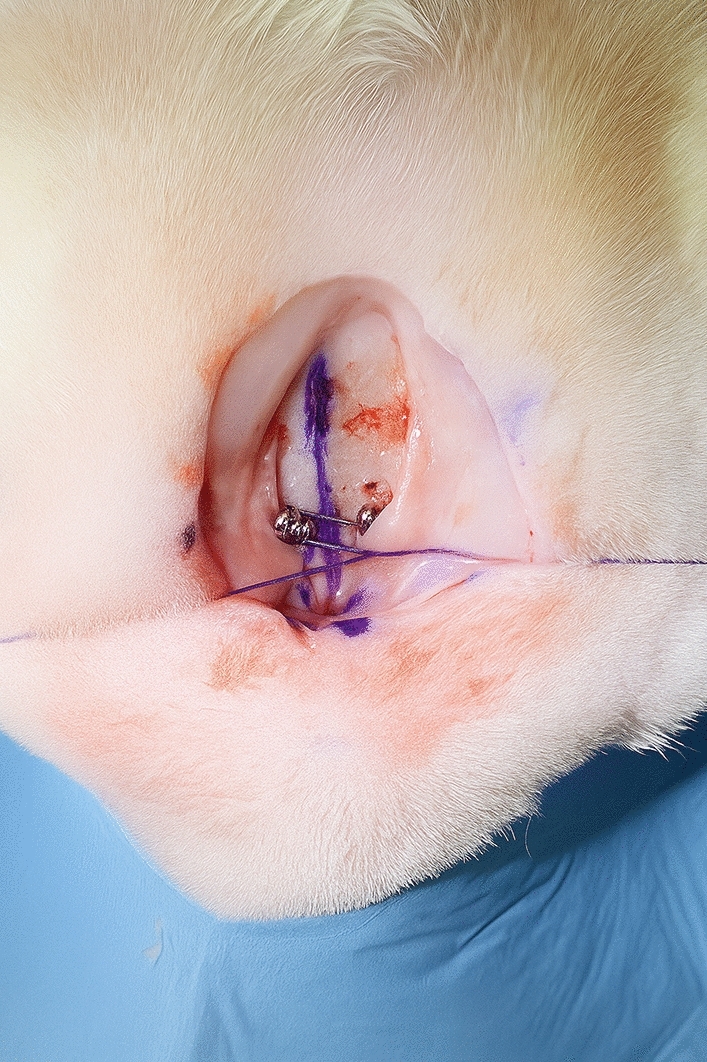
Digitally illustrated schematic image showing fixation of scalp flaps to the calvarium using cortical screws following subperiosteal dissection in Group 3

*Group 3 (Subperiosteal dissection)* After dissection, sharp periosteal release was performed along the orbital rims and nasal radix. Following midline skin excision (1 cm), a 4 mm auto-drive titanium screw was placed on each side of the cranial midline, and flaps were fixated with 3-0 polyglactin sutures. The subcutaneous tissue and skin were closed with 4-0 polyglactin and 5-0 polypropylene, respectively (Fig. 4).

All subjects were followed for 12 weeks postoperatively. At the end of this period, all animals were euthanized, and the surgical sites were excised in full thickness from periosteum to skin. For each subject, half of the samples were allocated to histological analysis and the other half to biomechanical testing, with allocation determined by randomization.

### Histologic Analysis

Tissue samples were fixed in 4% formalin, processed, and embedded in paraffin. Sections 5 µm thick were obtained from FFPE tissues and stained with hematoxylin–eosin (HE) and Masson’s trichrome. Stained sections were examined under a computer-assisted light microscope (DM 4000B, Leica, Germany), and images were captured using Leica Las V4.9 software. HE staining was used for morphological evaluation, and Masson’s trichrome for assessing collagen fiber distribution and localization. Bone tissue was excluded to focus on soft tissue architecture; the periosteum was identified by anatomical location and distinct histological features. Using ImageJ software (NIH, USA), the thicknesses of the epidermis (e), dermis (d), subcutaneous muscle (scm/SMAS), and periosteum (p) were measured from six randomly selected areas. Ratios between these thicknesses were calculated, and averages were determined to allow standardized intergroup comparison independent of absolute specimen size variation.

### Biomechanical Analysis

**Fig. 5:**
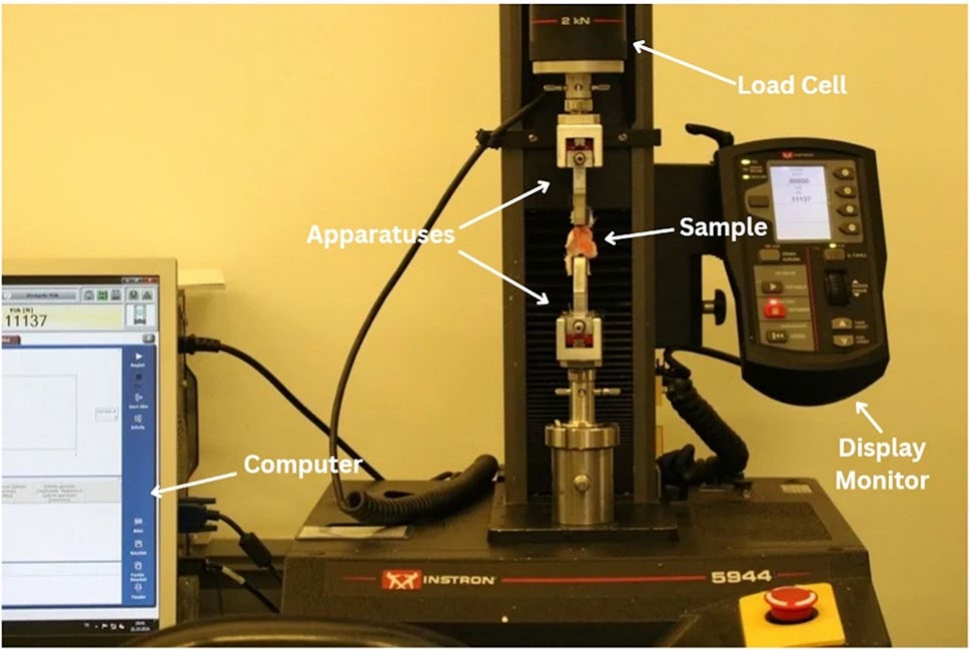
Schematic representation of the biomechanical test apparatus and its main components used in the tensile test

**Fig. 6:**
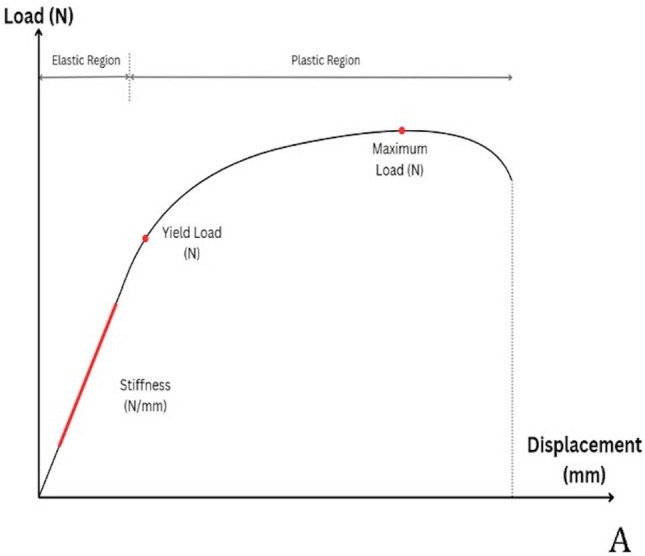
Graphical representation of the biomechanical parameters analyzed in the study. The sloped initial portion of the curve indicates stiffness, the point where the linear portion ends represents yield load, and the peak point represents maximum load. Elastic and plastic deformation regions are clearly delineated

All samples underwent static tensile testing using an Instron 5944 (Instron, Norwood, MA, USA) at a rate of 6.35 mm/min [R1]. The apparatus was designed to secure the samples and separate the stitched area (Fig. 5). Testing continued until failure, with load and displacement data recorded throughout. From load–displacement curves, maximum load (*N*), stiffness (N/mm), and yield load (*N*) were determined. Maximum load represented the ultimate load resisted, stiffness indicated resistance to deformation, and yield load marked the onset of permanent deformation. An illustration of these parameters is shown in Fig. 6.

Data from histological and biomechanical analyses were statistically compared among groups.

### Statistical Analysis

All statistical analyses were performed using IBM SPSS Statistics (Version 27, IBM Corp., Armonk, New York, USA). The Shapiro-Wilk normality test was applied to assess the distribution characteristics of the data. After confirming that the data followed a normal distribution, one-way analysis of variance (ANOVA) was used for intergroup comparisons. To determine intragroup differences following ANOVA, the Tukey post-hoc test was applied. A *p* value of <0.05 was considered statistically significant.

## Results

### Postoperative Course

In Group 1, two animals were lost due to respiratory distress; in Group 3, one animal was lost due to general health deterioration. New subjects were added to maintain group sizes. No wound-related complications occurred during follow-up.

### Histological Findings

The control group was evaluated as reference tissue, showing intact epidermis, dermis, subcutaneous fat, SMAS, and periosteum layers with hair follicles present. In all groups, occasional dermal detachment was seen as a processing artifact.

Histological sections from experimental groups also showed preserved layers with intact hair follicles. No necrosis or inflammatory infiltration was observed at 12 weeks. Masson’s trichrome staining revealed similar collagen fiber density and distribution across groups, with no evidence of increased density, architectural disorganization, or fibrotic scar tissue.

According to the quantitative measurements:

Epidermis/dermis ratio was 0.019 ± 0.013 in Group 1 (subcutaneous), 0.017 ± 0.005 in Group 2 (subgaleal), 0.019 ± 0.005 in Group 3 (subperiosteal), and 0.04 ± 0.026 in the control group. A statistically significant difference was found between the control group and Groups 2 (subgaleal) and 3 (subperiosteal) (*p* < 0.05).

Dermis/muscle (SMAS) ratio was 8.82 ± 3.27 in Group 1 (subcutaneous), 7.54 ± 1.86 in Group 2 (subgaleal), 7.21 ± 2.41 in Group 3 (subperiosteal), and 7.38 ± 3.78 in the control group. No statistically significant difference was found among the groups (*p* > 0.05).

Muscle (SMAS)/periosteum ratio was 0.92 ± 0.59 in Group 1 (subcutaneous), 0.96 ± 0.035 in Group 2 (subgaleal), 1.31 ± 0.47 in Group 3 (subperiosteal), and 2.11 ± 1.61 in the control group. No statistically significant difference was found among the groups (*p* > 0.05).

**Fig. 7:**
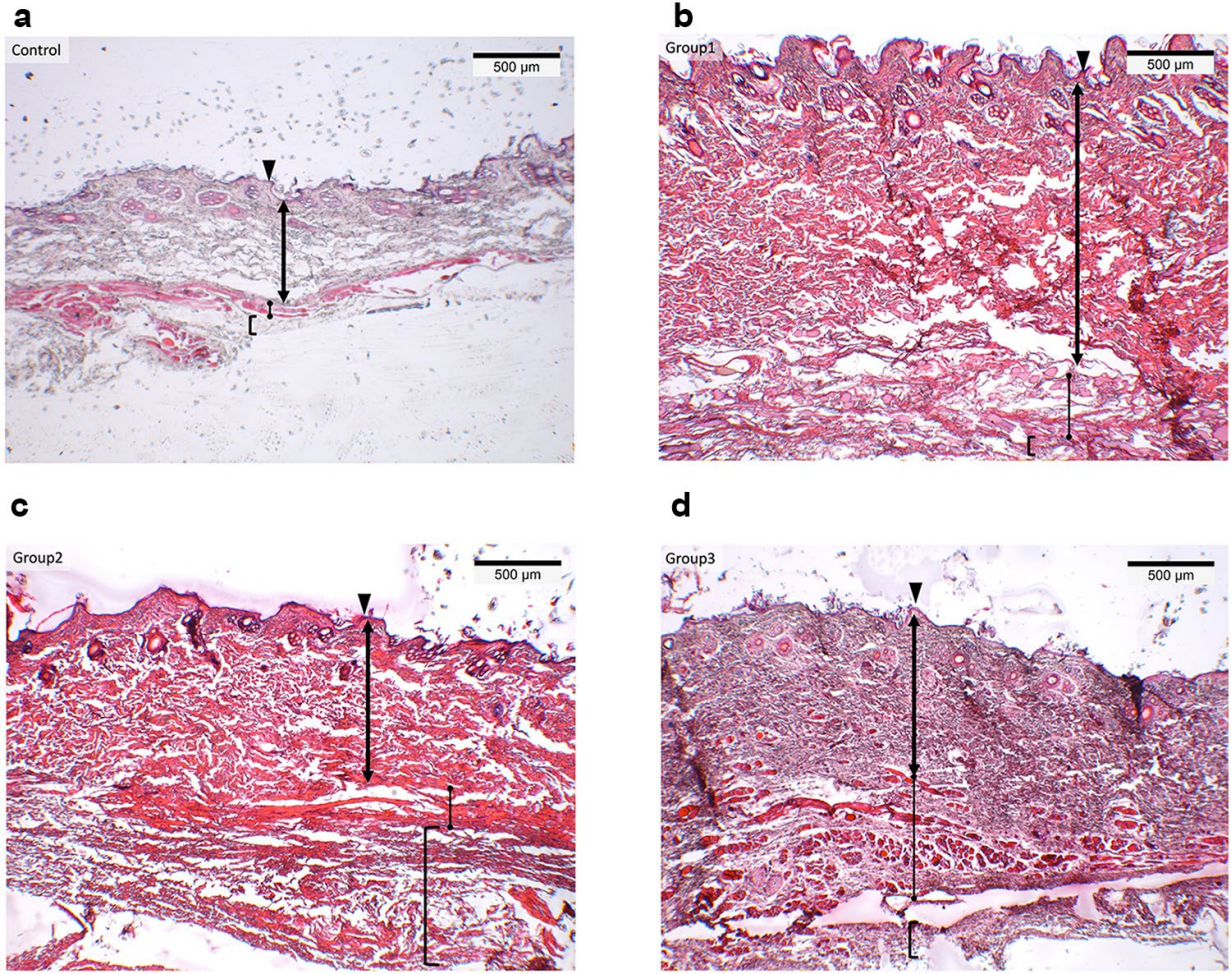
Hematoxylin and eosin (H&E)-stained histological sections from the Control group (**a**), Group 1 (**b**), Group 2 (**c**), and Group 3 (**d**). The epidermis (▲), dermis (double-headed arrow), SMAS (•), and periosteum ([) layers are clearly distinguishable. Scale bar: 500 µm

**Fig. 8:**
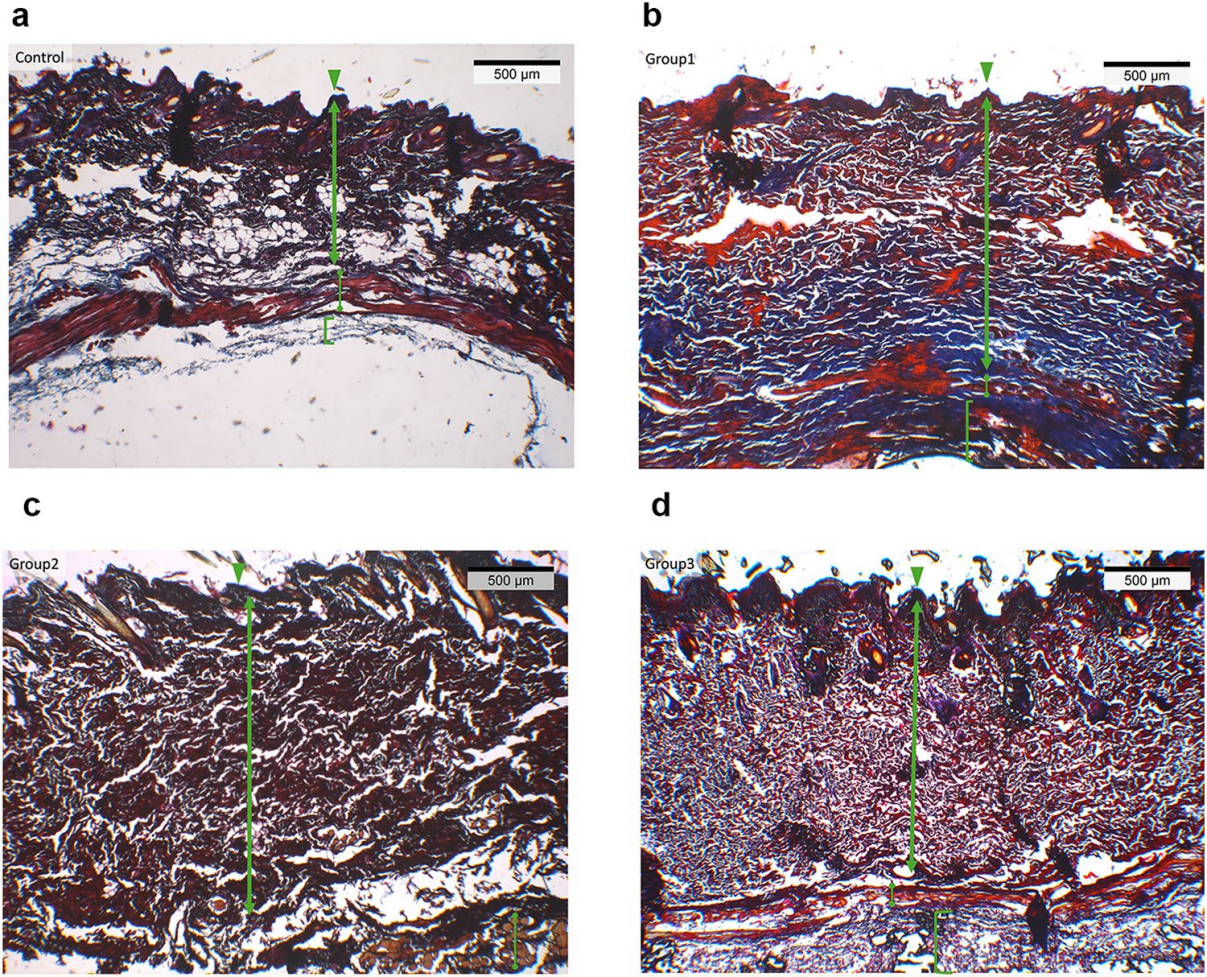
Masson’s trichrome-stained histological sections from the Control group (**a**), Group 1 (**b**), Group 2 (**c**), Group 3 (**d**). The epidermis (▲), dermis (double-headed arrow), SMAS (•), and periosteum ([) layers are clearly identified. Collagen fibers appear blue-green, muscle fibers are red, and nuclei are stained dark. Scale bar: 500 µm

Dermis/periosteum ratio was 8.6 ± 4.65 in Group 1 (subcutaneous), 6.38 ± 2.45 in Group 2 (subgaleal), 9.74 ± 6.58 in Group 3 (subperiosteal), and 16 ± 8.62 in the control group. Although the mean values in the experimental groups were lower than in the control group, the differences were not statistically significant (*p* > 0.05) (Figs. 7 and 8).

### Biomechanical Findings

**Fig. 9:**
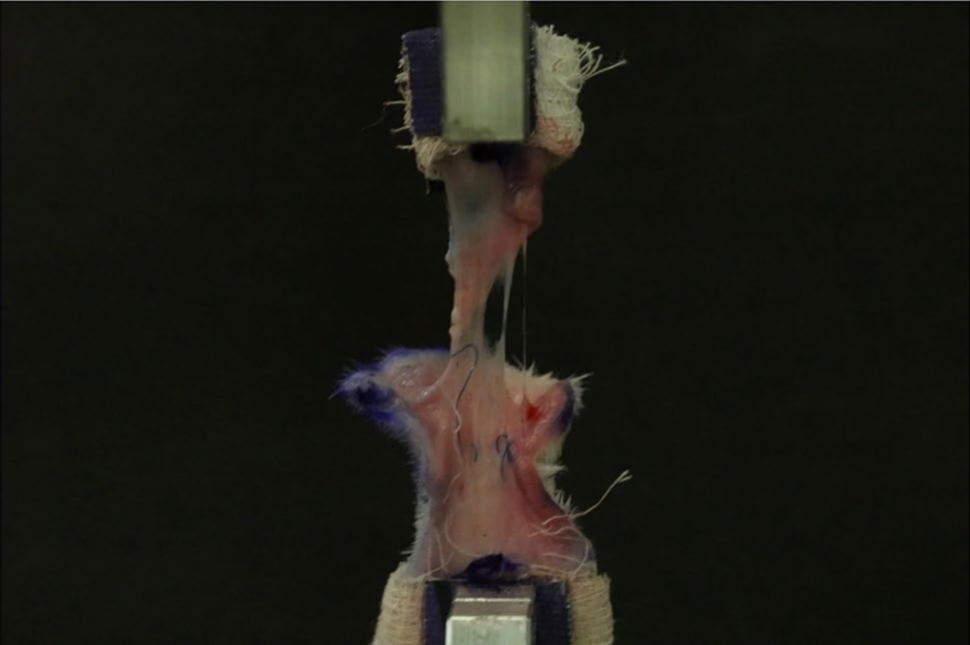
Image showing mechanical elongation and tissue separation of the specimen during the tensile test. The sample is fixed to the grips of the test device and is seen to elongate under maximum load application

All samples underwent tensile testing (Fig. 9) to separate soft tissue layers between the periosteum and skin. Mean ± SD values are shown in Table 1; statistical comparisons are in Table 2.

**Table 1:** The mean and standard deviation values of biomechanical parameters for each group

		Maximum load (N)	Stiffness (N/mm)	Yield load (N)
Control	Mean±SD	6.69±2.24	0.64±0.04	5.99±2.6
Group 1 (subcutaneous)	Mean±SD	16.87±6.09	2.14±0.81	14.56±7.37
Group 2 (subgaleal)	Mean±SD	11.44±3.0	2.56±0.86	9.10±3.11
Group 3 (subperiosteal)	Mean±SD	30.4±8.74	5.2±2.16	27.16±10.37

*SD* standard deviation

**Table 2:** Comparative analysis of biomechanical parameters among the groups

	Maximum load (N)	Stiffness (N/mm)	Yield load (N)
Control versus Group 1 (subcutaneous)	0.042*	0.026*	0.118
Control versus Group 2 (subgaleal)	0.058	0.010*	0.200
Control versus Group 3 (subperiosteal)	0.004*	0.012*	0.015*
Group 1 (subcutaneous) versus Group 2 (subgaleal)	0.121	0.482	0.173
Group 1 (subcutaneous) versus Group 3 (subperiosteal)	0.035*	0.033*	0.081
Group 2 (subgaleal) versus Group 3 (subperiosteal)	0.002*	0.035*	0.006*

*: *p *< 0.05/One-way ANOVA test

In summary, the subperiosteal group demonstrated the highest biomechanical performance across all parameters (maximum load: 30.4 ± 8.74 N; stiffness: 5.20 ± 2.16 N/mm; yield load: 27.16 ± 10.37 N). The subcutaneous group showed intermediate values (maximum load: 16.87 ± 6.09 N; stiffness: 2.14 ± 0.81 N/mm; yield load: 14.56 ± 7.37 N), followed by the subgaleal group (maximum load: 11.44 ± 3.00 N; stiffness: 2.56 ± 0.86 N/mm; yield load: 9.10 ± 3.11 N). The control group demonstrated the lowest values in all biomechanical parameters. Overall, the subperiosteal plane demonstrated statistically superior mechanical strength compared with both experimental planes and the control group, whereas the subcutaneous plane showed significantly greater resistance than the control group but lower values than the subperiosteal plane.

### Maximum Load

The highest maximum load value was observed in Group 3 (subperiosteal) with 30.4 ± 8.74 N. This was followed by Group 1 (subcutaneous) with 16.87 ± 6.09 N, Group 2 (subgaleal) with 11.44 ± 3.00 N, and the control group with 6.69 ± 2.24 N. Statistically significant differences (*p* < 0.05) were found in pairwise comparisons between the control group and Group 1 (subcutaneous), the control group and Group 3 (subperiosteal), Group 1 (subcutaneous) and Group 3 (subperiosteal), and Group 2 (subgaleal) and Group 3 (subperiosteal).

### Stiffness

In terms of rigidity, the highest mean value was observed in Group 3 (subperiosteal) with 5.20 ± 2.16 N/mm. This was followed by Group 2 (subgaleal) with 2.56 ± 0.86 N/mm, Group 1 (subcutaneous) with 2.14 ± 0.81 N/mm, and the control group with 0.64 ± 0.04 N/mm. Statistically significant differences (*p* < 0.05) were found in pairwise comparisons between the control group and all experimental groups, between Group 1 (subcutaneous) and Group 3 (subperiosteal), and between Group 2 (subgaleal) and Group 3 (subperiosteal).

### Yield Load

In terms of yield load, the highest mean value was again observed in Group 3 (subperiosteal) at 27.16 ± 10.37 N. This was followed by Group 1 (subcutaneous) with 14.56 ± 7.37 N, Group 2 (subgaleal) with 9.10 ± 3.11 N, and the control group with 5.99 ± 2.60 N. Statistically significant differences were found only between the control group and Group 3 (subperiosteal), and between Group 2 (subgaleal) and Group 3 (subperiosteal) (*p* < 0.05).

## Discussion

This experimental study provides a significant contribution to the literature as the first systematic model to conduct a comparative analysis—at both histological and biomechanical levels—among the three principal dissection planes commonly used in brow lift surgery: subcutaneous, subgaleal, and subperiosteal. Although numerous experimental studies in the current literature focus on the biomechanical advantages and histological healing of the subperiosteal plane, particularly regarding bone-periosteum integrity, the subcutaneous plane has often been excluded, and the effects of dissection on more superficial tissues have largely been overlooked [13, 19–23]. The distinctive feature of this study lies in its provision of quantitative histological evaluation encompassing all soft tissue layers, along with separate biomechanical analysis performed for each dissection plane directly subjected to surgical manipulation.

Histopathological examinations revealed that none of the dissection planes resulted in excessive fibrosis, chronic inflammation, necrosis, or architectural disruption at the 12-week endpoint. Although quantitative differences among groups were limited, this finding should not be interpreted as a null result. On the contrary, preservation of layered anatomy across all planes indicates that deeper tissue release does not inherently predispose to pathological scar formation. Therefore, the absence of histologic deterioration suggests that the biomechanical differences observed among planes are not driven by abnormal fibrosis, but rather by intrinsic healing mechanics and differences in tissue reattachment characteristics. Masson’s trichrome staining showed no significant differences among the groups in terms of collagen fiber density or distribution. Furthermore, no histological signs indicating fibrotic scar formation were observed. These findings suggest that the different dissection planes are biologically well tolerated and do not pose a risk of excessive wound healing or pathological scarring.

In the quantitative histological analyses, only the epidermis/dermis ratio was found to be significantly higher in the control group compared to the subgaleal and subperiosteal groups. This difference may have resulted from a slight reduction in epidermal thickness or remodeling associated with the healing process in the dermis. However, the absence of histological evidence of fibrotic scar tissue suggests that this change does not reflect a pathological wound healing issue and is unlikely to lead to a clinically significant complication. No statistically significant differences were observed between groups for the other ratios (dermis/SMAS, SMAS/periosteum, and dermis/periosteum). This indicates that despite the application of different dissection planes, the deeper tissue layers maintained similar thickness and organization, and the surgery did not cause any lasting alterations in tissue architecture. These findings are consistent with studies by authors such as Romo and Brodner, who reported that periosteal reattachment to bone is completed within 6–12 weeks in animal models, although such conclusions were not supported by quantitative histological data in those studies [18, 19]. Similarly, in Rapp’s rat model, it was reported that by day 28, the periosteum reattached to the bone through loose ligamentous structures, and by day 56, there was an increase in periosteal thickness accompanied by the formation of organized scar tissue [24]. In an experimental study conducted by Kim using a rabbit model, signs of healing such as periosteal inflammation, histiocytic activity, and new bone formation became prominent between days 8 and 12, and by day 17 had reached levels comparable to the control group [20]. These findings support the fully completed healing pattern observed at the end of the 12-week follow-up in our study, indicating that tissue integrity is biologically reorganized and stabilized across all dissection planes.

The biomechanical test results demonstrated that the subperiosteal dissection plane provided significant superiority in terms of maximum load (30.4 ± 8.74 N), rigidity (5.2 ± 2.16 N/mm), and yield load (27.16 ± 10.37 N). These findings are consistent with experimental data from researchers such as Kim, Kriet, and Thomas, who reported an increase in adhesion strength of the subperiosteal plane over time [13, 20, 21]. In Kriet’s study, it was reported that the avulsion force of the subperiosteal flap reached the same level as the control group by day 8; similarly, in Kim’s rabbit model, shear stiffness reached control levels by day 8, and ultimate shear strength by day 12 [20, 21]. Although Thomas reported in his study with up to 10 weeks of follow-up that the subgaleal plane provided greater flap strength in the early period, the same study showed that the tension curve of the subperiosteal flap increased more steeply over time, suggesting that it may offer more stable outcomes in the long term [13]. Indeed, studies focusing on early fixation data and limited follow-up durations may have led to the perception that subgaleal dissection is more advantageous. However, our observations indicate that the subperiosteal plane exhibits a slower but progressively developing adhesion process over time. When sufficient healing time is allowed, this plane appears to yield superior long-term stability. This hypothesis is strongly supported by the 12-week mechanical data obtained in our study. Clinically, increased maximum load and stiffness may indicate greater resistance to tissue recoil and gravitational descent, suggesting improved long-term positional stability of brow elevation when adequate fixation and healing time are provided. It should be acknowledged that screw fixation was applied in the subperiosteal group to simulate common clinical practice in endoscopic brow lift procedures. One may argue that rigid fixation could reduce early recoil forces and thereby influence scar remodeling. However, biomechanical testing in our study was performed after a 12-week healing period, when periosteal reattachment and tissue integration are expected to be biologically stabilized. At this stage, measured mechanical strength predominantly reflects intrinsic tissue adhesion rather than temporary fixation support. Furthermore, fixation in the subperiosteal group was intentionally incorporated to replicate real-world surgical conditions, as subperiosteal elevation without fixation does not reflect standard clinical technique.

The subgaleal plane has been evaluated in the literature regarding both its advantages and limitations. In Lindsey’s study, subgaleal dissection was reported to be superior to the subperiosteal plane in terms of avulsion force during the early postoperative period (days 2–4); however, by days 8 and 10, this finding had reversed, with the subperiosteal plane demonstrating stronger adhesion. Although the study lacked a control group and thus did not allow for reference-based comparison, it was noted that connective tissue began to organize around day 10 and that the inflammatory response diminished. These findings support the notion that subperiosteal flaps develop a more stable adhesion pattern over time and become mechanically more reliable in the later stages [22].

The subcutaneous plane has regained interest in recent years, particularly with the growing popularity of modern techniques such as the gliding brow lift. Researchers such as Viterbo, Savetsky, and Orra have reported that this dissection plane is effective in addressing superficial wrinkles, with associated low complication rates, minimal surgical trauma, and high patient satisfaction [4, 5, 25]. Subcutaneous dissection offers practical advantages as it does not require specialized equipment or necessitate osseous fixation. Additionally, leaving behind a thinner and lighter flap after dissection contributes to reduced tension across the fixation line.

The fibrotic scar tissue that develops as a result of disrupting the gliding plane through dissection allows for stronger adhesion of the brow to its new position during the healing process. However, in individuals with excessively lax, thick, or sebaceous skin, the long-term stability of elevation achieved through this plane may be questionable. Conversely, in patients with thinner skin or laxity limited to the superficial fascial layers or the skin itself, subcutaneous dissection may provide high predictability and satisfactory aesthetic outcomes. Supporting these clinical observations, the histological findings of the subcutaneous dissection plane also suggest a more physiological healing pattern.

In this study, although subcutaneous dissection demonstrated lower mechanical strength compared to the subperiosteal plane, it exhibited a histological profile most similar to the control group, particularly in the epidermis-to-dermis ratio. The differences observed were subtle and most likely reflect minor variations in superficial tissue remodeling rather than a clear biological advantage. One possible explanation for this finding is that subgaleal and subperiosteal dissections require deeper tissue release, which may transiently alter dermal thickness through mild edema, tension redistribution, or delayed remodeling, whereas subcutaneous dissection leaves these deeper support structures largely undisturbed. Because our analysis was ratio-based, we cannot determine whether the change resulted from reduced epidermal thickness or increased dermal thickness, and physiological variability in hair-bearing rabbit skin may also contribute. Validation of these findings in human or more human-like models, such as porcine scalp, would help clarify their clinical relevance. All three planes preserved normal architecture without fibrosis, indicating that the healing patterns were broadly comparable. It is important to distinguish mechanical stability from aesthetic surface appearance. Although histologic architecture appeared comparable among groups, the significant differences in maximum load, stiffness, and yield load indicate that resistance to tissue recoil and long-term positional stability may differ between planes. Brow lift durability depends not only on superficial scar quality, but also on the depth and strength of tissue adherence. Therefore, similar histological appearance should not be automatically interpreted as identical long-term mechanical performance or equivalent clinical durability.

One of the most notable aspects of our study is the exclusion of the calvarial bone in biomechanical testing. In classical studies conducted by Romo and Brodner, bone-periosteum composites fixed to the testing apparatus were evaluated [18, 19]. However, in our protocol, this approach was not preferred due to certain methodological issues that could compromise the test’s accuracy. When rigid structures such as the rabbit calvarium are fixed to the test apparatus, torque forces generated during tensile loading may obscure the true separation force. Moreover, in composite tissue samples, the point of rupture often occurs within the bone rather than at the soft tissue–periosteum interface, thereby reducing the reliability of the data in evaluating soft tissue strength. For this reason, our study focused solely on biomechanical evaluation of the soft tissue layers that are directly mobilized in clinical practice (epidermis, dermis, SMAS, and periosteum). The periosteal layer was further assessed histologically to compensate for this limitation, qualitatively. In this way, the study aimed to establish a model that not only reflects realistic clinical conditions, but also ensures greater methodological consistency.

### Limitations

First, although the rabbit model provides controlled insight into soft tissue healing and mechanical properties, rabbits exhibit faster healing, a lower fibrotic response, and different tissue composition compared with humans. Moreover, the use of young, healthy animals may further enhance regenerative capacity and reduce fibrotic remodeling relative to aging human brow tissues. Therefore, extrapolation to older aesthetic patients should be interpreted with caution.

Second, although a 12-week endpoint allows assessment of stabilized periosteal reattachment and scar maturation in rabbits, it may not fully capture long-term remodeling under dynamic facial stresses in humans. Human brow tissues are continuously subjected to multidirectional forces generated by animation, gravity, and soft tissue recoil.

Third, only static tensile testing was performed. While this permits standardized comparison of maximum load, stiffness, and yield load, it does not replicate cyclic or fatigue loading conditions encountered clinically. Future studies incorporating dynamic mechanical testing would enhance translational relevance.

In addition, physiological variability in rabbit skin thickness and composition may have influenced histological and biomechanical measurements despite standardized surgical technique and randomization.

Furthermore, because histological outcomes were expressed as proportional ratios rather than absolute layer thicknesses, it is not possible to determine whether the observed differences were driven by changes in epidermal or dermal thickness, or by combined remodeling effects.

Despite these limitations, the controlled design and direct comparison of three dissection planes provide meaningful comparative experimental data that may inform surgical plane selection.

## Conclusion

This experimental study demonstrated that different dissection planes in brow lift surgery lead to distinct biomechanical and generally similar histological outcomes. The subperiosteal plane provided superior mechanical strength, while the subcutaneous plane preserved a more physiological healing architecture. These findings support a tailored approach to plane selection, based on the patient’s anatomical characteristics, skin quality, and long-term surgical goals, recognizing that additional factors such as dynamic facial animation, vascularity, and individual healing capacity can influence clinical outcomes.
